# TLR3 Signaling Promotes the Induction of Unique Human BDCA-3 Dendritic Cell Populations

**DOI:** 10.3389/fimmu.2016.00088

**Published:** 2016-03-14

**Authors:** Nicholas J. Colletti, Hong Liu, Adam C. Gower, Yuriy O. Alekseyev, Christopher W. Arendt, Michael H. Shaw

**Affiliations:** ^1^Sanofi Pharmaceuticals, Cambridge, MA, USA; ^2^Department of Biological Science, Seton Hall University, South Orange, NJ, USA; ^3^Clinical and Translational Science Institute, Boston University, Boston, MA, USA; ^4^Department of Pathology and Laboratory Medicine, Boston University, Boston, MA, USA

**Keywords:** dendritic cell, TLR3, ILT3, ILT4, BDCA-3

## Abstract

Conventional and plasmacytoid dendritic cells (cDCs and pDCs) are the two populations of DCs that can be readily identified in human blood. Conventional DCs have been subdivided into CD1c^+^, or blood dendritic cells antigen (BDCA) 1 and CD141^+^, or BDCA-3, DCs, each having both unique gene expression profiles and functions. BDCA-3 DCs express high levels of toll-like receptor 3 and upon stimulation with Poly I:C secrete IFN-β, CXCL10, and IL-12p70. In this article, we show that activation of human BDCA-3 DCs with Poly I:C induces the expression of activation markers (CD40, CD80, and CD86) and immunoglobulin-like transcript (ILT) 3 and 4. This Poly I:C stimulation results in four populations identifiable by flow cytometry based on their expression of ILT3 and ILT4. We focused our efforts on profiling the ILT4^−^ and ILT4^+^ DCs. These ILT-expressing BDCA-3 populations exhibit similar levels of activation as measured by CD40, CD80, and CD86; however, they exhibit differential cytokine secretion profiles, unique gene signatures, and vary in their ability to prime allogenic naïve T cells. Taken together, these data illustrate that within a pool of BDCA-3 DCs, there are cells poised to respond differently to a given input stimulus with unique output of immune functions.

## Introduction

As key regulators of the immune system, antigen-presenting cells (APCs) play a primary role in acquiring, processing, and presenting both foreign and self-antigens to naïve CD4^+^ and CD8^+^ T cells, thereby initiating the adaptive branch of the immune system. Even though a variety of immunocytes, such as macrophages, monocytes, and B cells, are capable of functioning as APCs to a certain extent, dendritic cells (DCs) are considered the primary APCs for the stimulation of naïve T cells (1). In mammals, DCs are often broadly divided into two major groups, myeloid or conventional DCs (referred hereafter as cDCs) and plasmacytoid DCs (pDCs). In the human blood, three distinct subsets of DCs have been identified by their differential expression of three surface markers blood dendritic cells antigen (BDCA) 1, BDCA-2, and BDCA-3. BDCA-1 DC, or cDCs are characterized by their surface expression of Lin^−^, CD11c^+^, CD1c^+^, HLADR^+^, and CD123^dim^. BDCA-2 DCs, or pDCs, are identified by their surface markers Lin^−^, CD123^+^, CD4^+^, and HLADR^+^. The recently identified BDCA-3 cDCs are characterized as Lin^−^, CD123^−^, CD11c^+^, CD1c^−^, and by the unique expression of the chemokine receptor XCR1 (2, 3). BDCA-1 DCs express toll-like receptors (TLRs) 2-6 and 9, while BDCA-3 DCs express high levels of TLR3. Consistent with this TLR expression profile, BDCA-1 and BDCA-3 DCs produce large amounts of IL-12 during antibacterial and anti-viral responses (4, 5). BDCA-3 DCs have also been shown to be highly efficient at cross-presentation of antigen to T cells, in particular necrotic antigens (3).

In addition to inducing pro-inflammatory responses, DCs are also capable of promoting tolerogenic responses. The tolerance-inducing potential of DCs is intimately linked to their maturation status. For example, TLR-activated primary human pDCs have been shown to induce T regulatory cells (Tregs) *in vitro* under certain stimulatory conditions. It has also been previously demonstrated that immature cDCs, under steady state, exhibit the ability to prime naïve T cells to differentiate into IL-10 producing Tregs (6). The result of such a priming event can be influenced by the cytokines the DCs secrete, as well as the expression of receptors known to promote tolerance. Previous studies have shown that DCs differentiated *in vitro* with IL-10 (DC–IL-10) express high levels of inhibitory immune receptors, in particular the immunoglobulin-like transcript (ILT) family of surface receptors (7). ILT receptors have been shown to promote the induction of CD4^+^ Tregs through DC priming (8, 9). Additionally, TLR or CD40 ligand (CD40L) matured cDCs primed naïve T cells to differentiate into effector T cells (10). This dichotomy for cDCs to exhibit both immune-stimulatory and regulatory function suggests the presence of potentially different subsets or that the contrasting functions of cDCs are dictated by their differentiation/maturation state.

In the present study, using highly enriched primary human DCs, we embarked on an effort to stimulate various human DC subsets, including BDCA-3 DCs, to examine the upregulation of known tolerogenic markers. Interestingly, we observed that Poly I:C-stimulated BDCA-3 DCs can be subdivided into different populations based on their surface expression of ILT3 and ILT4. Our analysis of various ILT-expressing BDCA-3 DCs revealed populations that exhibit differential cytokine secretion profiles. In addition, gene expression profiling by microarray analysis revealed unique gene signatures for each population. Lastly, these different ILT-expressing populations of BDCA-3 DCs differ in their ability to prime effector T cells.

## Materials and Methods

### Isolation and Culture of Human Cells

Total blood leukapheresis was purchased from Research Blood Components LLC (Brighton, MA, USA). Total peripheral blood mononuclear cells (PBMCs) were isolated after lysis of red blood cells. Total DCs were first enriched using the Human Myeloid DC Enrichment Kit (StemCell Technologies, Vancouver, BC, Canada) according to the manufacturer’s instructions. Enriched DCs were then stained antibodies, including lineage markers (Lin) (BD Bioscience, San Jose, CA, USA), HLADR (BD Bioscience, San Jose, CA, USA), CD1c (Biolegend, San Diego, CA, USA), CD11c (Miltenyi, San Diego, CA, USA), CD123 (BD Bioscience, San Jose, CA, USA), and CD141 (Miltenyi, San Diego, CA, USA). Labeled cells were sorted on a BD FACS ARIA II (BD Biosciences, San Jose, CA, USA). pDCs were sorted based on the expression of cell surface markers Lin^−^, CD123^+^, and HLADR^+^. cDCs were sorted based on cell surface markers as Lin^−^, CD123dim, HLADR^+^, CD1c^+^, and CD11c^+^. BDCA-3 DCs were sorted based on cell surface markers as Lin^−^, CD123dim, HLADR^+^, CD1c^−^, and CD141^+^. The purity of collected pDCs, cDCs, and BDCA-3 DCs was consistently greater than 98% based on post sort analysis.

### RT-PCR for TLR Gene Expression (mRNA)

Total RNA was extracted from a total of 1 × 10^6^ freshly purified pDCs, cDCs, and BDCA-3 DCs utilizing the RNeasy Plus Mini kit (Qiagen, Valencia, CA, USA). The RNA was reversely transcribed to cDNA utilizing SuperScript VILO (Invitrogen, Grand Island, NY, USA). TLRs 1–10 expression was analyzed using Applied Biosystem’s TaqMan Gene Expression Master Mix and primer/probes. PCR parameters were 50°C for 2 min, followed by 95°C for 10 min proceeding to 40 cycles at 95°C for 15 s, 60°C for 1 min. RPLPO was used as an internal control. Taqman assays were performed on a BioRad Real-Time PCR System CFX384 (Biorad, Hercules, CA, USA). To determine the relative expression of each gene, the 2^−ΔΔCt^ approach (ΔCq method) was employed (11).

### Gene Array Experiments

Purified BDCA-3 DCs were cultured in complete X-VIVO-15 (5% human serum (Sigma) + 1% Pen/Strep (Invitrogen, Grand Island, NY, USA)) media containing 10 μg/mL Poly I:C at 37°C for 18 h. Cells were washed and sorted by the expression of ILT3 and ILT4 (R&D Systems, Minneapolis, MN, USA). Total RNA was extracted from ILT3^−^ ILT4^−^, ILT3^+^ ILT4^−^, ILT3^+^ ILT4^+^, and ILT3^−^ ILT4^+^ BDCA-3 DCs utilizing the RNeasy Plus Mini kit (Qiagen, Valencia, CA, USA). RNA was frozen and sent to the Boston University MicroArray Core for further processing.

All procedures were performed at Boston University Microarray Resource Facility as described in GeneChip^®^ Whole Transcript (WT) Sense Target Labeling Assay Manual (Affymetrix, Santa Clara, CA, USA), Nugen Ovation Pico WTA System User Guide, Nugen WT-Ovation Exon Module User Guide, and Nugen Encore Biotin Module User Guide (Nugen, San Carlos, CA, USA).

### Microarray Analysis

Affymetrix GeneChip Human Gene 1.0 ST CEL files were normalized to produce gene-level expression values using the implementation of the robust multiarray average (RMA) (12) in the affy Bioconductor R package (version 1.36.1) (13) included in the Bioconductor software suite (version 2.12) (14) and an Entrez Gene-specific probeset mapping (version 17.0.0) from the Molecular and Behavioral Neuroscience Institute (Brainarray) at the University of Michigan (15). Array quality was assessed by computing relative log expression (RLE) and normalized unscaled standard error (NUSE) using the affyPLM Bioconductor package (version 1.34.0). Principal component analysis (PCA) was performed using the prcomp R function with expression values that were unadjusted or were adjusted for donor (by creating linear models using the lmFit function in the limma package (version 3.14.4), treating donor as a fixed effect) and had then been normalized across all samples to a mean of 0 and a SD of 1. Linear mixed-effects modeling and the associated analysis of variance were carried out using the anova.lme function in the nlme package (version 3.1-97). Pairwise differential gene expression was assessed by performing Student *t*-tests on the coefficients of linear models created using lmFit, correcting for donor as a fixed effect. Correction for multiple hypothesis testing was accomplished using the Benjamini–Hochberg false discovery rate (FDR). All microarray analyses were performed using the R environment for statistical computing (version 2.15.1).

### Identification of Differentially Expressed Genes

For comparative analysis, general linear models for microarray data were performed for probe sets present on the microarray to identify probe sets that were differentially expressed between the groups, based on moderated t-statistics. Probe sets with a 1.5-fold change and a *P*-value <0.05 were considered biologically significant. PCA was then performed. PCA is a mathematical transform that collapses the variance between samples across a set of large set of variables (here, all ~20,000 genes on the array) into a much smaller set of variables called principal components (PCs). These “meta-variables” are arranged such that PC1 explains the most variance in the data, followed by PC2, etc. PCA was performed using all genes across all samples, either before or after adjusting the expressions for donor (using a simple linear model), and plots were made of PC1 vs. PC2.

### BDCA-3 DC Activation Assay Followed by ILT4 Sorting

After confirming the post sort purity of >98%, 1 × 10^5^ BDCA-3 DCs were plated in 96 well v-bottom plate in complete X-VIVO 15 media (Lonza). TLR agonists, Poly I:C, and LPS (Invivogen, San Diego, CA, USA) were added at a final concentration of 10 μg/mL. Plates were incubated at 37°C for 18 h. Cells were then harvested and stained with ILT4 (R&D Systems, Minneapolis, MN, USA). After staining, BDCA-3 DCs were sorted based on their expression of ILT4 for subsequent analysis.

### Cytokine Production Assays

Immediately following sorting, total BDCA-3 DCs were added to a FACS tube containing Poly I:C at a final concentration of 10 μg/mL in complete X-VIVO 15 media (5% human serum, 2 mM l-glutamine, and 1× pen/strep). BDCA-3 DCs were incubated at 37°C for 18 h to allow for the formation of ILT4-positive cells. Stimulated BDCA-3 DCs were then re-sorted by their expression of ILT4 directly into complete X-VIVO 15 media without further stimulation. 2 × 10^4^ cells in 200 μL complete X-VIVO 15 media were added to a 96 well plate and incubated for 18 h at 37°C. Supernatants were harvested and cytokine profiles were assayed with the ProcartaPlex Human Cytokine/Chemokine/Growth Factor Panel (eBiosciences, San Diego, CA, USA) on a Bioplex 200 System running Bioplex Manager Version 6. Statistics were performed by running a two-tailed paired student *t*-test in GraphPad Prism version 6.0. Results with a *P*-value <0.05 were considered significant. Intracellular FACS staining of BDCA-3 DCs was performed as follows. Following sorting, total BDCA-3 DCs were added to a FACS tube containing Poly I:C at a final concentration of 10 μg/mL in complete X-VIVO 15 media (5% human serum, 2 mM l-glutamine, and 1× pen strep). BDCA-3 DCs were incubated at 37°C for 18 h to allow for the formation of ILT4 positive cells. Golgistop was then added for 6 h. Cells were then washed and surface stained with ILT3, ILT4 (R&D Systems), and CD141 (Miltenyi). Following surface staining, cells were stained with Live/Dead (Life Technologies) as per the manufacturer’s protocol. Finally, cells were intracellularly stained with IFN-γ (BioLegend), IL-4 (eBiosciences), IL-10 (BD), IL-13 (BD), IL-5 (BioLegend), and TNFα (BioLegend). Data were collected using BD LSRII and analysis was performed with FlowJo Software V9.7 (Treestar).

### *In vitro* Priming of Naïve CD4^+^ and CD8^+^ T Cells

An aliquot of total PBMC was enriched using Human Pan T cells Pre-Enrichment Kit (StemCell Technologies, Vancouver, BC, Canada) for the preparation of allogenic naïve CD4^+^ and CD8^+^ T cells. Total CD4^+^ and CD8^+^ T cells were stained and FACS sorted on a BD FACS ARIA II. Naïve T cells were designated as CD25^−^, CD127^+^, CD62L^+^, and CD49d^low^. BDCA-3 DCs were isolated as stated previously. Bulk BDCA-3 DCs were incubated at 37°C for 18 h with 10 μg/mL Poly I:C. After TLR stimulation, BDCA-3 DCs were sorted into pure populations of ILT4^+^ and ILT4^−^. Allogenic naïve CD4^+^ and CD8^+^ T cells were incubated with allogenic BDCA-3 DCs (ILT4^+^ and ILT4^−^) at a 1:5 DC to T cell ratio at 37°C in complete X-VIVO 15 media. After 7 days, primed T cells were harvested and analyzed for cell surface phenotype as well as intracellular staining.

### Flow Cytometry

Blood dendritic cells antigen-3 DC activation status was assessed using surface stain markers CD40, CD80, CD86, and CCR7 from BD Biosciences. Cells were stained with fluorescent antibodies for 30 min on ice, washed twice with BD FACS staining buffer (DPBS contains 2% FBS and 0.09% sodium azide) and then acquired on a BD LSR II. Mean fluorescence intensity and cell percentages were determined by FlowJo V9.7 (Treestar). Cytokine production by day 7 primed T cells was assessed by intracellular cytokine staining (ICS) after initial staining with Cell Trace violet to detect proliferation (Life Technologies). Day 7 primed T cells were stimulated with PMA (50 ng/mL; EMD Millipore) and Ionomycin (1 μM; Sigma, St. Louis, MO, USA) in the presence of Golgi Stop (1 μl/mL; BD Bioscience) for 6 h. After incubation, cells were first surface stained with antibodies to CD25 (BD) and HLA-G (BioLegend), 30 min on ice. Next, cells were cultured using Live/Dead stain (Life Technologies) according to manufacturer’s protocol. Finally, cells were stained intracellularly with antibodies against IL-4 (eBiosciences) and IFN-γ (BD). Data were collected using BD LSRII and analysis was performed with FlowJo Software V9.7 (Treestar).

## Results

### TLR3 Signaling Results in the Formation of Several BDCA-3^+^ DC Populations

**Figure 1:**
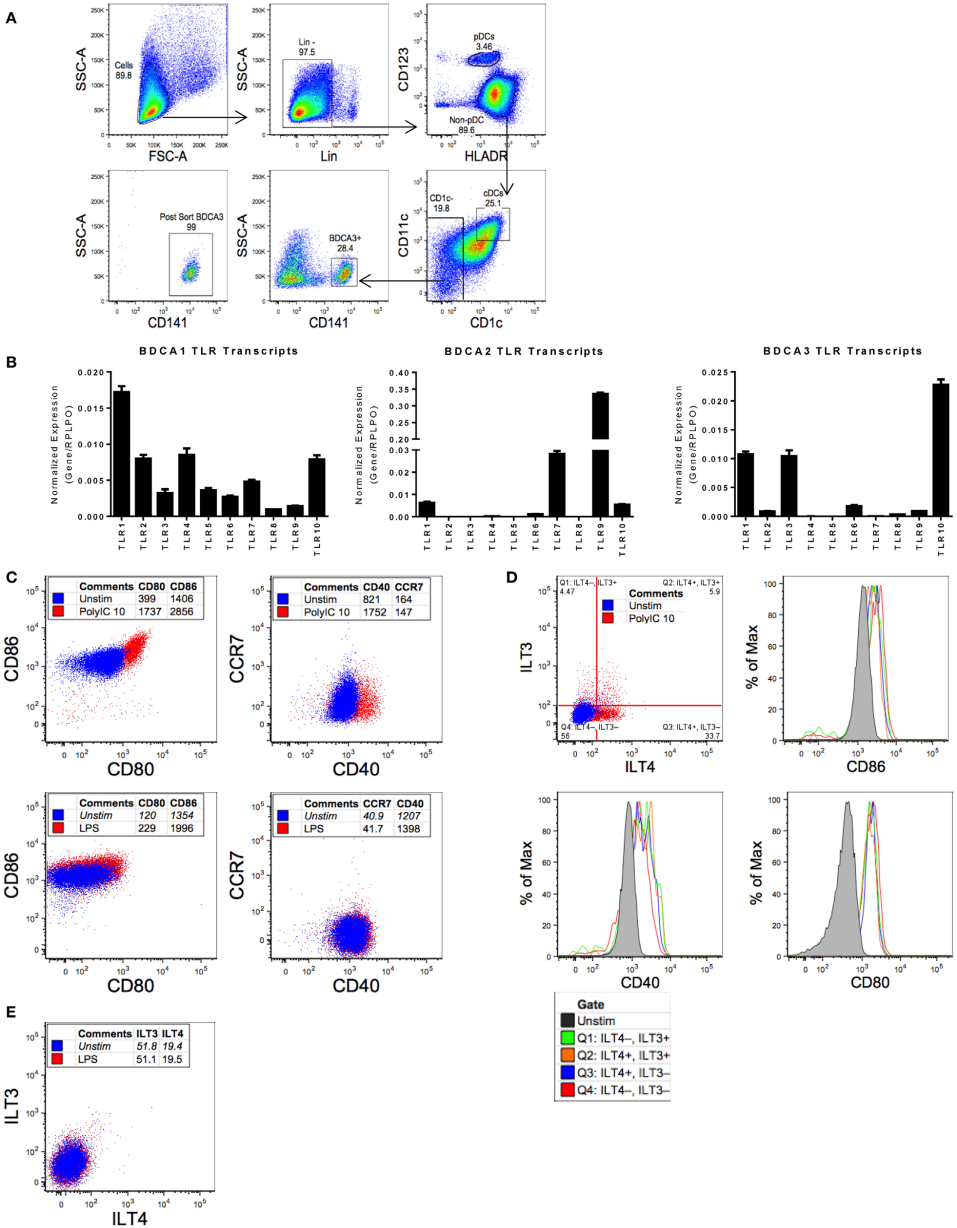
**Purified BDCA-3 cDCs upon stimulation with a TLR3 agonist yield multiple populations as measured ILT3 and ILT4 expression**. **(A)** Gating strategy for sorting human blood dendritic cells (as described in Section “Materials and Methods”). **(B)** RNA extracted from sorted pDCs, cDCs, and BDCA-3 cDCs was reverse transcribed for qPCR. To determine the relative expression of each gene of interest, normalized to RPLPO, the 2^−ΔΔCt^ approach (ΔCq method) was utilized. Figure represents four donors. **(C)** BDCA-3 cDCs were stimulated with Poly I:C and LPS at 10 μg/mL for 18 h. Expression of CD80, CD86, CCR7, and CD40 comparing pre- and post-stimulation, blue dots represent unstimulated cells and red dots identify cells stimulated with TLR agonist, with values representing mean fluorescent intensity (MFI). **(D,E)** BDCA-3 cDCs stimulated with 10 μg/mL Poly I:C **(D)** or LPS **(E)** for 18 h, ILT3 and ILT4 population’s CD40, CD80, and CD86 expression compared by MFI. Data shown are one representative donor out of four.

Isolating sufficient numbers of highly enriched pDCs and cDCs for functional assays requires a large number of input PBMCs. pDCs and cDCs only make up approximately 0.1–0.3% of total PBMCs in healthy individuals, respectively (16). A two-step enrichment process was devised to facilitate the isolation of sufficient quantities of both cDCs and pDCs from a leukapheresis pack. First, total blood DCs were enriched by negative selection, followed by flow cytometry sorting of the enriched DC fraction for pDCs (Lin^−^, CD123^+^, HLADR^+^), cDCs (Lin^−^, CD123^−^, HLADR^+^, CD1c^+^, CD11c^+^), and BDCA-3 cDCs (Lin^−^, CD123^−^, HLADR^+^, CD1c^−^, CD141^+^) (Figure 1A). Previous studies suggested that primary cDCs and pDCs exhibit distinct patterns of TLR expression (17) as compared to *in vitro* monocyte-derived DCs (18, 19). We, therefore, wanted to confirm, as previously reported, the TLR expression profile of highly purified pDC and cDC populations to ascertain their phenotype prior to TLR agonist profiling. qPCR on the sorted primary blood DCs revealed that cDCs expressed a wide range of TLRs, including TLR1, TLR2, TLR4, and TLR10. By contrast, pDCs mainly expressed TLR 7 and 9, and BDCA-3 cDCs expressed mainly TLR1, TLR3, and TLR10 (Figure 1B). Given that BDCA-3 cDCs expressed a limited repertoire of TLRs, we focused our initial efforts to characterize the BDCA-3 cDC response to Poly I:C, a TLR3 agonist.

The robust expression of TLR3 transcript in BDCA-3 cDCs suggests that this population of cDCs is particularly responsive to TLR3 agonists. We decided to evaluate BDCA-3 cDC’s response to Poly I:C, a TLR3 agonist. Stimulation of BDCA-3 cDCs with Poly I:C resulted in activation of these DCs as indicated by the induction of canonical DC-maturation markers, such as CD40 and CD80/86 (Figure 1C). Poly I:C-induced BDCA-3 DC maturation was both dose and time dependent (data not shown). Interestingly, concomitant with the Poly I:C-induced maturation of BDCA-3 cDCs was the appearance of distinct populations, which can be distinguished based on the differential expression of ILT3 and ILT4 (Figure 1D). The appearance of these populations (ILT3^−^ ILT4^−^, ILT3^−^ ILT4^+^, ILT3^+^ ILT4^−^, and ILT3^+^ ILT4^+^) of BDCA-3 cDCs was not only dose dependent but also occurs with rapid kinetics following 18 h of stimulation with Poly I:C (data not shown). To discount the possibility that the various ILT populations arose due to differential levels and/or threshold of activation, the maturation status of each of the ILT populations was examined. As shown in Figure 1D, all ILT populations had similar surface expression of DC-maturation-associated markers. To address the specificity of the inductive signal for generating these various populations of BDCA-3 cDCs, the TLR4 agonist LPS was added to purified BDCA-3 cDC cultures for 18 h. As shown in Figure 1E and consistent with the lack of TLR4 transcripts in BDCA-3 cDCs, TLR4 triggering did not promote the upregulation of ILT3 and ILT4. These data suggest that the inductive signal driving the development of these populations, designated by the expression of ILT3 and ILT4 on BDCA-3 cDCs, is not due to the *in vitro* culturing conditions but rather specific to TLR3-mediated signaling.

### ILT4^+^ BDCA-3 cDCs Have Unique Cytokine Profiles and Genomic Signatures Compared to ILT4^−^ Cells

**Figure 2:**
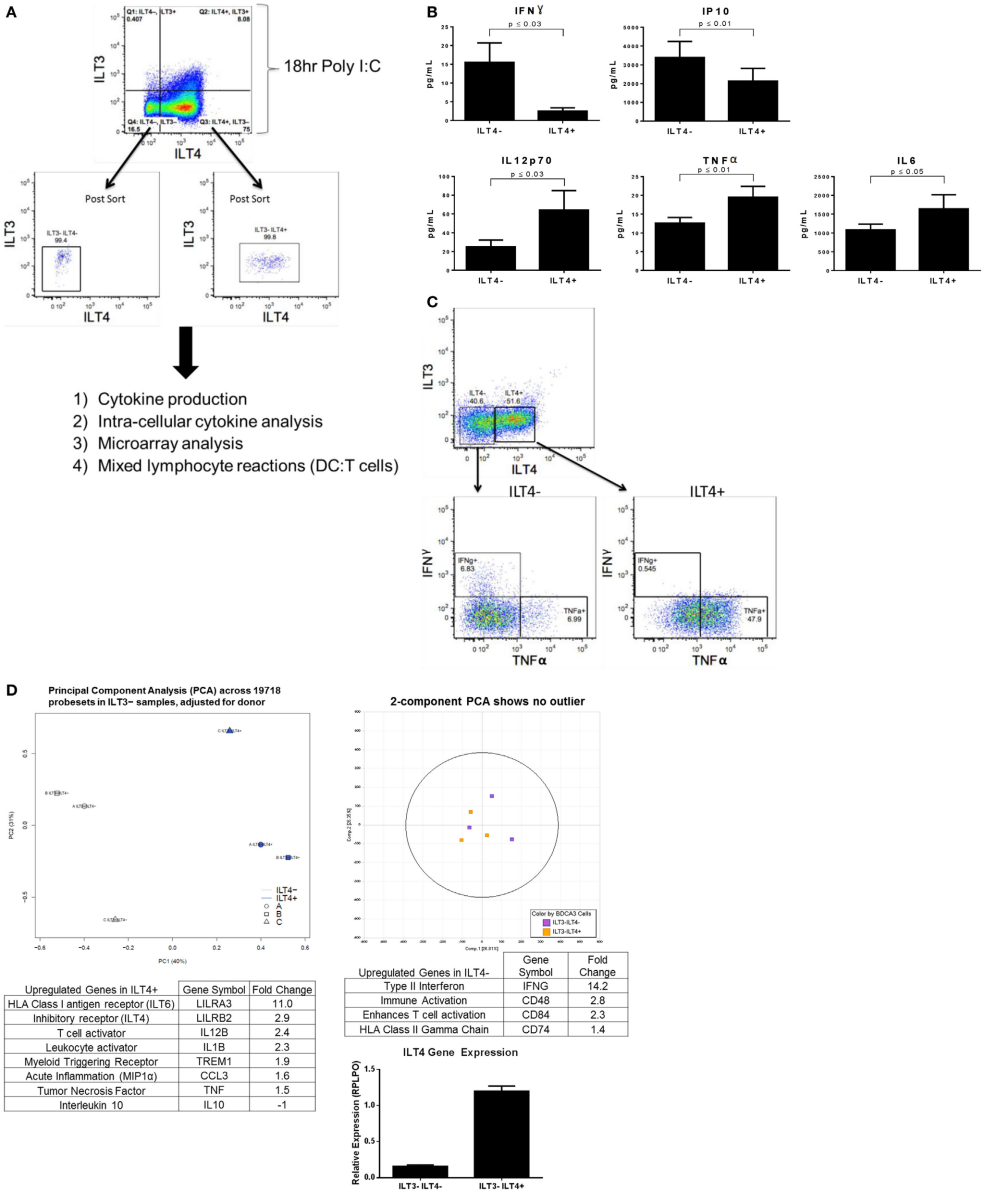
**Poly I:C-stimulated ILT4^−^ and ILT4^+^ BDCA-3 cDCs have unique cytokine and gene signatures**. **(A)** Experimental design of BDCA-3 DC phenotyping. **(B)** BDCA-3 cDCs were cultured with Poly I:C for 18 h and then sorted into ILT4^−^ and ILT4^+^ populations. Cells were then plated without further stimulation for 18 h. Supernatants were assayed for cytokine and chemokine content by luminex analysis. *P*-values generated using two-tailed student’s paired *t*-test (95% confidence interval). Graphs represent four donors. **(C)** BDCA-3 cDCs were stimulated with Poly I:C for 18 h, Golgistop was then added for 6 h. Cells were surface stained with ILT3, ILT4, and CD141 and then intracellularly stained with IFN-γ and TNF-α. Data showing intracellular staining are representative of one donor out of four. **(D)** Genomic profiling of ILT4^−^ vs. ILT4^+^ was performed using GeneChip Human Gene 1.0 ST arrays. Principal component analysis (PCA) was computed using OmicSoft ArrayStudio, and a plot was generated to show the relative clustering of ILT4^−^ and ILT4^+^. ILT4^−^ and ILT4^+^ populations were compared to each other by *t*-test with a threshold set for a fold change >1.5 and a *P*-value <0.05. ILT4 gene expression was confirmed by qPCR. (Data shown are one representative donor out of four).

Following stimulation of BDCA-3 DCs with Poly I:C, the emergence of several populations designated by their expression of ILT receptors, which have suggested inhibitory effects, prompted us to investigate whether these populations of the TLR3-induced DCs also exhibit differential cytokine production. Due to limited cell numbers in the ILT3^+^ILT4^+^ population, we focused our initial investigative efforts on the cytokine secretion profile of the ILT4-expressing DCs (experimental design; Figure 2A). To determine the level of cytokine secretion among the ILT4^−^ vs. ILT4^+^ populations, bulk-sorted BDCA-3 DCs were stimulated with Poly I:C for the induction of ILT4^±^ cells. After 18 h, ILT4^−^ and ILT4^+^ populations were FACs-sorted and re-cultured overnight in the absence of further TLR stimulation. As demonstrated in Figure 2B, multiplex cytokine analysis of the cultured supernatant revealed quantitative and qualitative differences in the cytokine secretion potential between the ILT4^+^ and ILT4^−^ populations. ILT4^−^ cells are unique in their capacity to produce IFN-γ and IP-10, while ILT4^+^ cells are poised for TNF-α, IL-12p70, and IL-6 production (Figure 2B). To confirm the unique cytokine-secreting profiles between ILT4^+^ and ILT4^−^ cells, we performed ICS by stimulating BDCA-3 DCs for 18 h with Poly I:C and assessed cytokine secretion by each population (Figure 2C). Consistent with the cytokine analysis, ICS analysis reveal that ILT4^−^ BDCA-3 DCs are capable of producing IFN-γ and low levels of TNF-α, conversely, ILT4^+^ BDCA-3 DCs produced exclusively high levels of TNF-α and undetectable levels of IFN-γ (Figure 2C). Taken together, the data thus far suggest that ILT4^−^ and ILT4^+^ BDCA-3 DCs are phenotypically and functionally unique.

To better understand whether the ILT4^+^ and ILT4^−^ populations represent DC populations with unique characteristics, transcriptional profiling was performed on ILT4^+^ vs. ILT4^−^ BDCA-3 DCs after Poly I:C stimulation. General linear models for microarray data were performed for probe sets present on the microarray to identify probe sets that are differentially expressed between the groups, based on moderated t-statistics. Probe sets with a 1.5-fold change and a *P*-value <0.05 were considered significant. Although our analysis revealed unique gene signatures for the ILT4^+^ vs. ILT4^−^ populations following stimulation, we were not able to identify unique surface markers to faithfully distinguish between the two populations. A 3D plot generated by PCA with OmicSoft ArrayStudio across all probe sets revealed that ILT4^+^ cells are most dissimilar from ILT4^−^ cells (Figure 2D). The ILT4^−^ population revealed upregulated genes involved in T cell stimulation, in particular IFN-γ, which is consistent with multiplex cytokine and ICS analysis (Figures 2B,C). Finally, the ILT4^+^ population revealed the upregulation of two inhibitory receptors, ILT4 and ILT6. The ILT4 expression of the ILT4^+^ population was confirmed by real-time PCR analysis (Figure 2D). The microarray analysis of the ILT4^+^ population showed upregulation in the TNF-α gene, these results are all consistent with both multiplex cytokine analysis and FACS intracellular staining (Figures 2B,C). Collectively, the functional and genomic analysis of ILT4^+^ and ILT4^−^ cells suggest that these cells are distinct DC populations found within the broader BDCA-3 DCs.

### ILT4^−^ and ILT4^+^ BDCA-3 DCs Differ in Their Ability to Prime Allogenic Naïve T Cells

**Figure 3:**
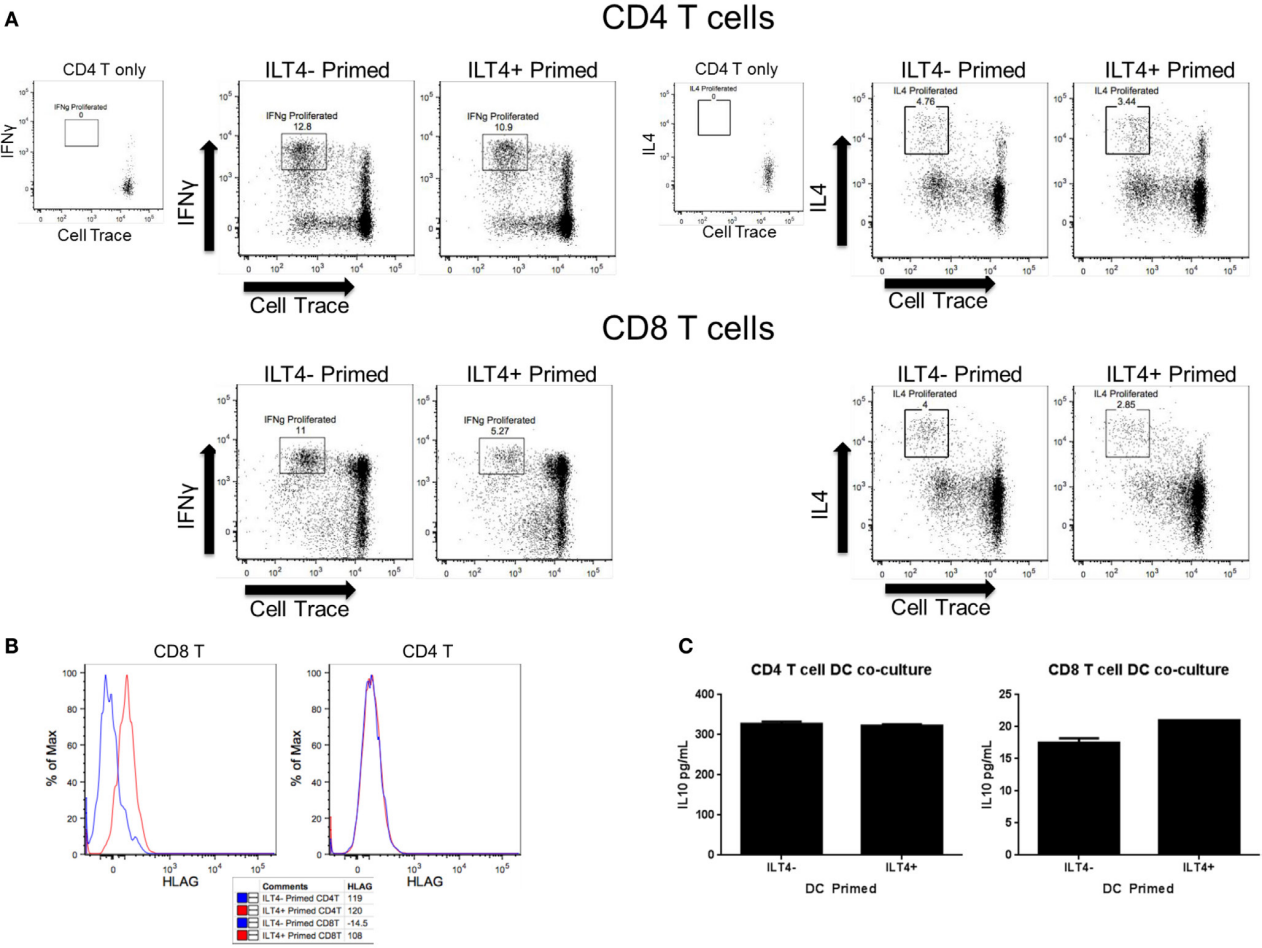
**ILT4^−^ and ILT4^+^ BDCA-3 cDCs have unique naïve T cell priming capabilities**. **(A,B)** Total enriched BDCA-3 cDCs were cultured with Poly I:C for 18 h. Cells were then sorted into ILT4^−^ and ILT4^+^ populations and incubated with either naïve CD4 or CD8 allogenic T cells (CD25^−^, CD127^+^, CD62L^+^, CD49d^low^). After 7 days, the resultant T cells were assessed for surface marker and intracellular cytokine expression. Dot plots are gated on live CD3^+^, CD4^+^, or CD8^+^ T cells. **(C)** IL-10 cytokine levels from day 7 T cell/DC co-culture supernatant. Data shown are one representative donor out of four.

The differences in cytokine secretion and transcriptional profiles of ILT4^−^ and ILT4^+^ BDCA-3 DCs imply that these DC populations may have distinct T cell priming potential. To address this possibility, we assessed the ability of each ILT4 population to prime naïve CD4^+^ and CD8^+^ T cells. To this end, BDCA-3 DCs were first stimulated with Poly I:C and subsequently sorted to high purity into ILT4^−^ and ILT4^+^ populations. The sorted ILT4 populations were then co-cultured with allogenic sorted naïve CD4^+^ and CD8^+^ T cells at a DC to T cell ratio of 1–5. T cells were stained with Cell Trace prior to the MLR reaction to assess the level of their proliferation. On day 7 post priming, the phenotype of the resultant CD4^+^ and CD8^+^ T cells was assessed by flow cytometry for the secretion of prototypic Th1- and Th2-associated cytokines, such as IFN-γ and IL-4, respectively. Both ILT4^+^ and ILT4^−^ DCs demonstrated the capacity to prime both naïve CD4^+^ and CD8^+^ T cells as demonstrated by the dilution of Cell Trace dye (Figure 3A). More importantly, both DC populations preferentially primed naïve CD4^+^ and CD8^+^ T cells toward IFN-γ^+^ Th1 cells (Figure 3A). IL-4-producing cells were also observed, but at much lower frequency than that of IFN-γ cells. Despite that ability to prime naïve CD4^+^ and CD8^+^ T cells toward the Th1 phenotype, ILT4^+^ DCs appeared to be less efficient at promoting Th1 induction as compared to ILT4^−^ DCs (Figure 3A). CD4^+^ T cell activation remained largely intact, while the priming of CD8^+^ T cells was impaired by as much as 53% when co-cultured with ILT4^+^ DCs (Figure 3A). To investigate a possible explanation for the impaired T cell priming potential of ILT4^+^ DC, we examined the expression of the human leukocyte antigen (HLA)-G, a putative ligand for ILT4 receptor (20). Cell surface expression of HLA-G has been implicated in the induction of tolerogenic functions in various physiological and pathological settings (21–24). Assessment of the T cell expression of HLA-G after priming with ILT4^+^ and ILT4^−^ DCs revealed that CD8^+^ T cells primed in the presence of ILT4^+^ DCs showed an approximately ninefold increase in HLA-G expression as compared to CD8^+^ T cells co-cultured with ILT4^−^ DCs (Figure 3B). On the other hand, there was no observable induction of HLA-G expression on CD4^+^ T cells co-cultured with either ILT4^+^ or ILT4^−^ DCs. The observed impaired T cell priming ability of ILT4^+^ DCs could be the result of active inhibition by suppressor CD8^+^ T cells expressing HLA-G. To this end, we investigated the level of IL-10, a known anti-inflammatory cytokine secreted by various cell types, including Tregs (25), in our DC primed T cell cultures. As shown in Figure 3C, we found similar levels of T cell-derived IL-10 in both ILT4-positive and -negative DCs primed T cell cultures. Taken together, these data suggest a potential IL-10-independent mechanism of dampening DC priming capabilities.

## Discussion

Our initial characterization of TLR expression pattern of a homogeneous highly purified human BDCA-3 DCs is consistent with previous studies (26), which demonstrated high TLR3 expression. The study of BDCA-3 DC biology has classically been associated with TLR3 agonists, namely Poly I:C. Interestingly, in addition to TLR3 expression, we observed that BDCA-3 DCs also exhibited high transcript levels for TLR1 and TLR10. TLR1 recognizes bacteria-associated peptidoglycan and lipoproteins in concert with TLR2 (27). Despite the low level of TLR2 gene expression (Figure 1B), stimulation with TLR1/2 agonist PAM3CSK4 consistently induced BDCA-3 DC activation (data not shown), indicating that BDCA-3 DCs express functional TLR1/TLR2 heteroreceptor complex capable of responding to lipoprotein. The high transcript level of TLR10 suggests that BDCA-3 DCs express functional TLR10 protein. However, given that the role of TLR10 in mediating immune function and its ligand have yet-to-be-determined precluded us from further characterize TLR10 function in BDCA-3 DCs. Transcript expression of various TLR receptors and responsiveness to multiple TLR agonists suggests two hypotheses: (A) on per cell basis BDCA-3 DCs express multiple TLRs or (B) there may exist various BDCA-3 DC subsets each with a unique TLR expression. The recent discovery of a human XCR1^+^CD141^+^ DC subset expressing TLR3 within the conventional DC population supports of the latter hypothesis (2). This discovery raises the possibility that multiple yet-to-be-identified populations of BDCA-3 DCs may exist.

Currently, BDCA-3 DCs can be identified and purified based on the absence of lineage marker expression (Lin^−^) and CD1c^−^, and the co-expression of HLADR and CD141. In the present study, we demonstrated that stimulation of BDCA-3 DCs with Poly I:C, a TLR3 agonist, induced the expression of canonical markers associated with DC activation/maturation, such as CD40, CD80, and CD86. Interestingly, Poly I:C stimulation also induced the expression of ILT3 and ILT4 as detected by flow cytometry. The expression pattern of the ILT receptors allowed for the distinction of various populations, namely ILT3^−^ ILT4^−^, ILT3^+^ ILT4^−^, ILT3^−^ ILT4^+^, and ILT3^+^ ILT4^+^, within the total BDCA-3^+^ population. ILT3 and ILT4 are surface proteins of the immunoglobulin superfamily, which have been demonstrated to be expressed on monocytes and DCs. The cytoplasmic region of ILT molecules contains a putative immunoreceptor tyrosine-based inhibitory motif, suggesting an inhibitory function of ILT receptors. Consistent with the proposed inhibitory function, ILT3 has been shown to induce immunosuppression, including T cell anergy, Treg induction, and reduced allo-stimulatory capacity (8, 9). The similar expression of activation markers within each of the described ILT populations (Figure 1D) led us to hypothesize the potential for functional differences among these various ILT3- and ILT4-expressing DC populations.

Previous studies have confirmed the expression of inhibitory ILT receptors on monocyte-derived BDCA-3 DCs, which were generated in the presence of various growth factors and cytokines, including IL-10. These DCs, termed DC–IL-10, expressed high levels of the receptors ILT2 and ILT3 (7). DC–IL-10 cells were shown to exhibit immunosuppressive features, such as high IL-10 production and generation of CD4^+^ Tregs (7). More recently, BDCA-3 DCs expressing high ILT3 levels have been identified in the dermis of human skin. These dermal ILT3^+^ BDCA-3 DCs are characterized by their capacity for constitutive IL-10 production, inducing T cell hyporesponsiveness, and inhibiting skin inflammation by the induction of Tregs (28). In addition to ILT3 expression, transcript profiling performed on primary blood BDCA-3 DC have also demonstrated the presence of the inhibitory receptor ILT4 following Poly I:C stimulation by gene array (29). Due to the consistently limited number of cell yield from ILT4^−^ILT3^+^ and ILT4^+^ILT3^+^ post Poly I:C stimulation, we focused our efforts on phenotyping ILT4^±^ILT3^−^ BDCA-3^+^ DCs. To investigate the potential of functional differences between ILT4^−^ and ILT4^+^ BDCA-3^+^ DCs, we began by comparing the transcriptional profiles of ILT4^−^ or ILT4^+^ DCs by microarray. From this analysis, we found unique transcripts that were differentially expressed between ILT4^−^ and ILT4^+^ DCs. Consistent with the high surface expression of ILT4, ILT4 transcript was increased in ILT4^+^ BDCA-3 DCs stimulated with Poly I:C (Figure 2D). ILT6 expression was also highest in ILT4^+^ BDCA-3^+^ DC samples. ILT6, unlike other ILT members, lacks a transmembrane domain and is a soluble receptor (30). As immune modulators, the heightened expression of ILT4 and ILT6 may work in synergy to attenuate T cells priming in ILT4^+^ DC MLR reactions (Figure 3). In addition to the enhanced transcript expression of ILT4 and ILT6, ILT4^+^ BDCA-3 DCs expressed higher levels of transcripts for interleukin-12 beta-subunit (IL-12b/IL-12p40) and tumor necrosis factor α (TNF-α). The expression of IL-12b, which is a shared subunit for both bioactive IL-12p70 and IL-23, suggests that ILT4^+^ BDCA-3 DCs may promote the generation of either T-helper 1 (Th1) and/or Th17 cells, respectively. Consistent with the IL-12b transcript levels, the production of bioactive IL-12p70 by ILT4^+^ BDCA-3 DCs was greater as compared to ILT4^−^ BDCA-3^+^ DCs. TNF-α is a well-studied pro-inflammatory cytokine. TNF-α is known for its ability to induce systemic inflammation and the acute phase reaction. TNF-α regulates the expansion and survival of CD4^+^ and CD8^+^ T cells and has been implicated in the progression of various diseases (31–33). In addition to IL-12b and TNF-α, ILT4^+^BDCA-3 DC produced exclusively CCL3, a chemokine that has been shown to enhance the differentiation, migration, and effector functions of CD8^+^ T cells (34). Taken together, ILT4^+^BDCA-3 DCs, based on their cytokine production, are functional to provide a link between innate and adaptive immunity.

ILT4^−^ BDCA-3 DCs expressed mainly type II interferon (IFN-γ). These results were confirmed at the protein level by both intracellular FCS and luminex analysis (Figures 2B,C). IFN-γ plays an important role in both innate and adaptive immunity. IFN-γ is involved in anti-viral, -bacterial, and -tumor biology (35–37). The capacity of ILT4^−^ BDCA-3 DCs to produce IFN-γ following stimulation is reminiscent of previously described interferon-producing killer dendritic cells (IKDC) (38). Murine IKDCs are distinct from cDCs and pDCs and with the molecular expression profile of both NK cells and DCs. They produce substantial amounts of IFN-γ and exhibit cytolytic capacity (39, 40). Whether ILT4^−^BDCA-3 DCs are the human equivalent/counter part of the murine IKDCs remains to be determined. In addition to IFN-γ, high levels of IP-10 were also detected in the supernatant of cultured ILT4^−^ BDCA-3 DCs. The high levels of IP-10 are probably due to the secretion of high levels of IFN-γ. IP-10 is known to induce the chemotaxis of various immunocytes including T cells (41, 42). ILT4^−^ BDCA-3 DCs also expressed several immune activators, including CD48, CD84, and CD74. CD84 is a cell surface receptor expressed on monocytes, macrophages, granulocytes, and DCs involved in leukocyte activation. CD84 function on myeloid cells remains unknown (43). CD74 is involved in the regulation of class II major histocompatibility complex (MHC) proteins in APCs. Taken together, the upregulation of these CD markers may contribute to ILT4^−^ DC’s ability to more robustly prime naïve CD4^+^ T cells (Figure 3A top panel) (44). Recent studies have confirmed the presence of a unique subset of BDCA-3 DCs, cells that are also XCR1^+^. XCR1 is a chemokine receptor for the ligand XCL1. In our microarray analysis, we did not detect any differences in the expression of XCR1 between the ILT4^−^ and ILT4^+^ populations, suggesting that XCR1 is not a contributing factor toward the phenotypes observed. Considering the differences in gene expression profiling, in particular the high expression of ILT receptors on ILT4^+^ BDCA-3 DCs and the high level of IFN-γ by ILT4^−^ BDCA-3 DCs, we propose that ILT4-BDCA-3 DCs extend the BDCA-3 DC family, representing a cell type possessing dual innate effector functions and antigen-presenting capacity.

Blood dendritic cells antigen-3 DCs have the ability to prime naïve T cells and influence their polarization toward various T-helper cell phenotypes, in particular toward Th1 priming (45). The location of such DCs also influences their capacity to prime toward a regulatory phenotype. Skin BDCA-3 DCs were shown to secrete high levels of IL-10 and induce highly potent Tregs (28). BDCA-3 DC–IL-10 showed high expression of ILT receptors. The high expression of ILT3 was shown to be involved in BDCA-3 DCs impaired allo-stimulatory capacity to prime naïve T  ells (7). In our study, we showed that stimulated primary human BDCA-3 DCs with Poly I:C generated several populations of ILT-expressing cells. In an allogenic MLR reaction, we showed that BDCA-3 DCs expressing high levels of ILT4 have a reduced allo-stimulatory capacity. This reduced capacity was more prominent for CD8^+^ T cell activation, yet negligible with CD4^+^ T cells. This is unanticipated, given that our analysis of both ILT4^−^ and ILT4^+^ BDCA-3 DCs suggests that these cells are potent T cells activators. The discrepancy in the capacity of ILT4^−^ and ILT4^+^ BDCA-3 DCs to prime T cells may be directly linked to the activity of the ILT4 receptor. Previous studies have determined that HLA-G is a natural ligand for ILT4 (46). HLA-G, a non-classical class I heavy chain, is typically expressed on fetal-derived placental cells and has been shown to inhibit allogenic MLRs (47, 48). We tested the expression of HLA-G on the resulting primed T cells after the MLR with BDCA-3 DCs. Interestingly, we observed an increased expression of HLA-G on primed CD8^+^ T cells co-cultured with ILT4^+^ DCs (Figure 3B). The expression of HLA-G on primed CD8^+^ T cells could be associated with the induction of a subpopulation of IL-10 producing suppressor T cells. However, IL-10 secretion was similarly observed in all cultured conditions. This suggests a possible suppressive mechanism that is IL-10-independent. This is consistent with previous studies demonstrating that HLA-G^+^ T cells present within PBMCs do not mediate suppression through IL-10 secretion (49). Nonetheless, the induced expression of HLA-G on T cells by a distinct DC population may represent an immune-dampening mechanism to prevent the overstimulation of the adaptive arm of the immune response. Taken together, these observations suggest that within a Poly I:C-stimulated population of bulk BDCA-3 DCs, there are cells poised for immune-stimulation, as well as to dampen the immune response.

Our study contributes to the already expanding knowledge of DC phenotypes. We have shown that a sorted culture of BDCA-3 DCs, as identified by our current understanding of phenotypic markers, actually consists of cells that respond differently to an activation stimulus (cytokine secretion, genomic analysis, and T cells priming). We have subdivided Poly I:C-stimulated BDCA-3 DCs by their expression of ILT4. They differ in their ability to secrete cytokines and prime naïve T cells. Through genomic analysis, we have shown that they have unique gene signatures associated with unique biological processes, such as T cell priming and inflammation. Taken together, these populations of BDCA-3 DCs may work in synergy to mediate different aspects of an ongoing immune response.

## Author Contributions

HL, AG, and YA provided microarray analysis. MS and CA provided experimental help and design. MS and NC wrote the manuscript. NC performed all the described experiments.

## Conflict of Interest Statement

The authors declare that the research was conducted in the absence of any commercial or financial relationships that could be construed as a potential conflict of interest.
